# New Smoothie Products Based on Pumpkin, Banana, and Purple Carrot as a Source of Bioactive Compounds

**DOI:** 10.3390/molecules27103049

**Published:** 2022-05-10

**Authors:** Marcin Kidoń, Pascaline Aimee Uwineza

**Affiliations:** 1 Department of Food Technology of Plant Origin, Poznan University of Life Sciences, 60-624 Poznan, Poland; 2 Department of Chemistry, Poznan University of Life Sciences, 60-628 Poznan, Poland; pascaline.uwineza@up.poznan.pl

**Keywords:** smoothie, phenolic compounds, anthocyanin, carotenoids, vitamin C, antioxidant activity, vegetable, fruit

## Abstract

Smoothies are fruit- and/or vegetable-based products in form of beverages that are typically semi-liquid, thick in consistency, and mainly consist of purees and juices. Other ingredients, such as yogurt, milk, ice cream, sugar, honey, or simply water may also be added. The present study aimed to elaborate smoothie products based on bananas, pumpkins, and purple carrots. These fruits and vegetables were chosen due to their high bioactive compounds content, potential health benefits, and availability to industry. Five smoothie formulations were produced and analyzed for pH, soluble solids, total phenolic content, anthocyanins, carotenoids, vitamin C, antioxidant activity, instrumental color, and sensory features. From the analysis, the result showed that the obtained smoothies were a good source of total phenolic content (39.1 to 55.9 mg/100 g) and anthocyanin (7.1 to 11.1 mg cyanidin-3-glucoside/100 g), and that they possessed high antioxidant activity (4.3 to 6.2 µM Trolox/g). From sensory evaluation, all the produced smoothies were desirable, but the formulations with banana were scored higher compared to the pumpkin. In conclusion, smoothies composed of pumpkin, banana, and purple carrot can be a good new food product that incorporates nutritional compounds into the human diet.

## 1. Introduction

Fruits and vegetables are an essential part of the human diet. In particular, they are rich sources of dietary fiber, vitamins, and various phytochemicals. Numerous studies have proved that they play a vital role in health promotion and prevention of certain chronic diseases, e.g., hypertension, cancer, coronary heart disease, stroke, etc. [1,2]. In addition to this, they provide a lot of sensory sensations. Fruit and vegetables are rich in colorful, flavorful, and tasteful compounds.

Banana is one of the earliest crops cultivated by man and remains a staple food crop for millions of people in the tropical world. Bananas are monocotyledonous plants, belonging to the genus *Musa* of the family Musaceae in the order Scitamineae. The majority of edible cultivars are allopolyploid triploids with a genome constitution of AAA (dessert banana), AAB (plantains), and ABB (cooking bananas) [3]. This fruit is mostly grown in hot, tropical climates across the globe, predominantly in Asia, Latin America, and Africa. The biggest producers are India, China, Indonesia, the Philippines, Ecuador, and Brazil. Bananas production in the world exceeds 100 million tons andit is the second highest produced fruit after citrus, accounting for around 16% of global fruit production [4]. Despite having a high calorie content primarily from carbohydrates and dietary fiber, bananas are poor in protein and fat. Vitamins C, A, B_1_, B_2,_ and B_6_, as well as minerals, such as magnesium, phosphorus, calcium, and iron could be also found in bananas. Banana consumption could be associated with a reduction in the risk of gastrointestinal diseases, regulation of carbohydrate metabolism, and weight control [5,6].

Carrot is a type of root vegetable. This plant is widely cultivated and consumed throughout the world, such as in America, Europe, Southwest Asia, and Africa. Its popularity is due to its versatile use, taste, and health benefits. The global production of carrots and turnips in 2019 was about 45 million tons. China produces about 50% of the world’s total carrots. Other meaningful producers of carrots are Uzbekistan, Russia, the USA, Ukraine, and Poland [4]. Purple carrot varieties are still popular, and these are traditionally grown in countries where carrots are originated, such as Turkey, Afghanistan, Egypt, Pakistan, and India. Cultivated and wild carrots belong to *Daucus carota* species. Cultivated carrots could be divided into the following two main groups: the eastern anthocyanin (*Daucus carota* ssp. sativus var. atrorubens Alef.), which are yellow or purple in color, and the western carotene (*Daucus carota* ssp. sativus var. sativus), which are yellow or orange. Indeed, carrot roots with purple and yellow colors are the ancestors of today’s more popular orange varieties. Carotenoids and phenolics are the major antioxidant compounds found in carrots. Alasalvar et al. [7] identified 11 phenolic acids in different colored carrot roots, but the total concentration of all phenolic acids was the highest in purple carrots. Purple carrots also contain twice the amount of alpha and beta carotene compared to orange carrots. The color of purple carrot roots comes from pigments called anthocyanins, which are a type of flavonoids. Anthocyanins from purple carrot are more stable than pigments from other sources, even during heating and processing, and also act as very powerful antioxidants [8,9,10,11]. Bioactive compounds found in purple carrot roots could play a vital role in preventing or delaying cardiovascular disease (CVDs), obesity, diabetes, and cancer. Anthocyanins and other phenolics are especially successful in reducing metabolic changes and possessed inflammatory effects, as well as preventing oxidative stress [12].

Pumpkins are defined as fruits of different species classified in the *Cucurbitaceae* family and genus *Cucurbita*, but they are regarded as a vegetable in consumer terms. Pumpkin originally came from Northeastern Mexico and the Southern United States, but nowadays this vegetable is spread across every continent. In 2019 global pumpkin production was about 23 million tons. Asian countries, such as China and India, are the biggest pumpkin producers in the world. The Russian Federation, Ukraine, and the United States are also significant pumpkin producers [4]. The most important cultivated species are *Cucurbita pepo*, *C. maxima*, *C. moschata*, *C. mixta*, and *C. stilbo*. Pumpkin fruits have a substantial variability in shape, size, and color of skin and flesh. Fruits, apart from food usage, also have decorative and agricultural properties. All parts of the fruit are eatable, but the most important are the flesh and seeds. It can be consumed raw, but the flesh is also suitable for the preparation of purees, jams, jellies, and pies, whereas the seeds can be consumed after drying or roasting, or processed into oil via pressing. Pumpkin fruits are a naturally rich source of dietary antioxidant vitamins like A, E, and C, carotenoids like β-carotene, lutein, and zeaxanthin, and phenolic compounds like caffeic acid, gallic acid, 4-hydroxybenzoic acid, protocatechuic acid, and rutin. Pumpkins can be also recognized as a source of dietary fiber, minerals, and unsaturated fatty acids. The content of particular compounds varies, and is affected by genotypic differences and growing conditions. Intake of these compounds can have a beneficial effect on human health and, as such, pumpkin could be considered as a “functional food” [13,14,15,16]. In the literature, the anti-diabetic, antioxidant, anti-carcinogenic, anti-inflammatory, and anti-microbial potential of pumpkin ingredients can be found. Also, anti-kidney stone formation, anti-hypotensive, anti-inflammatory, and anti-blood-coagulatory effects are well documented [17].Most fruits and vegetables are consumed fresh. However, many of them are industrially processed into canned, dried, juiced, frozen, or soup products to extend their shelf life, protect their nutritional value, and make meal preparation easier. Nowadays, consumer trends are orientated to ready-to-eat and functional food, and/or the reformulation of typical products to increase nutritional value. Smoothies could fulfill this consumer demand [1]. Smoothies are new products on the market, and are potentially a convenient and palatable way to replace at least one portion of fruit or vegetables from the recommended five portions per day. Smoothies are usually semi-liquid, thick beverages, obtained by blending fruit, fruit juice, and/or fruit puree. To increase sensory sensation, water, ice, sugar, sweeteners, spices, seeds, yogurt, or milk can be added. In order to increase the quantity of bioactive compounds in smoothies, some researchers proposed incorporating fruit seeds and peels into smoothies, as these are generally wasted during processing, or selecting raw materials that are rich in phytochemicals for inclusion in smoothies [18,19,20]. However, it must be considered that these products also contain considerable amounts of simple sugars. Excessive consumption of simple carbohydrates, particularly from low-dietary fiber products like fruit juices, could be associated with the metabolic syndrome or obesity. Because of this, it is recommended to consume not more than one portion per day. This is especially important for children and individuals at risk of obesity or type 2 diabetes, who should limit their consumption of simple sugar-containing products [21,22].

The phytochemical contents and sensory properties of smoothies can vary substantially depending on the processing methods utilized, as well as the raw ingredients used during the preparation [19]. Therefore, it is always advised to carefully select the processing method and raw materials in order to produce a food product with high nutritional values and desirable sensory features. The main objectives of this study were the development of new smoothie formulations based on the available nutritious ingredients, including bananas, pumpkins, and purple carrots, as well as to investigate bioactive compound contents, sensory characteristics, antioxidant activity, and other quality parameters of the obtained products.

## 2. Results and Discussion

### 2.1. pH and Soluble Solids

The pH and total soluble solids results of the five produced smoothies, pumpkin and purple carrot with high sugar (PCH), pumpkin and purple carrot with low sugar (PCL), banana and purple carrot with low sugar (BCL), banana and purple carrot without sugar (BCW), and banana, pumpkin, and purple carrot without sugar (BPCW) are presented in Table 1. The results showed that the pH of the produced smoothies ranged between 3.91 and 4.45, and that soluble solids ranged between 7.9 and 16.7%. The pH and soluble solids were different due to different ingredient ratios and different ingredient acidity and sugar content. BCL and BPCW smoothies had the highest pH level of 4.45, while PCH showed the lowest pH level of 3.91. The pH values of food products are important for sensory characteristic, as well as for the prevention of microbial spoilage. These pH values were typical for smoothies and similar to other findings. For example, a smoothie obtained by mixing juçara, banana, and strawberry pulps has a pH of about 4.0, and a smoothie consisting of pineapple, watermelon, banana, and coconut milk possessed a pH of about 4.5 [23,24]. According to other studies, the main ingredients for smoothie production used in this work showed different acidity. Purple carrot has a pH of about 6.2, bananas range between 4.8–5.4, pumpkin range between 4.4–4.5, and lemon has a pH of about 2.3 [16,25,26,27,28]. It has been claimed that sour fruit juices could successfully replace currently used artificial acidity regulators, especially in functional food production [28]. Lemon juice added to smoothies decreased pH effectively.

The total soluble solids are related mainly to the sugar concentration in the product. The BCL smoothie had the highest total soluble solids (16.7%), followed by the BCW (12.7%) while the PCL smoothie had the lowest total soluble solids (7.9%, Table 1). The result showed that there was a large difference in soluble solids values in the produced smoothies. The formulations which contained banana had almost two times the soluble solids values of those with pumpkins and carrots only. Aditionally, Uzodinma et. al. [24] noticed that an increased amount of bananas in smoothie formula decreased moisture content. According to different research, banana itself has a higher total soluble solid content compared to pumpkins and purple carrots. Ribeiro et al. [29] reported 23.1 Brix in banana pulp, and Wang et al. [5] reported 14.73 Brix in banana smoothies. Witrowa-Rajchert et al. [30] reported 15.2% dry matter content in fresh purple carrots cv. Deep Purple. Additionally, Quitȃo-Teixeira et al. [31] reported 8.21 Brix in carrot juice, and Zinash et al. [32] reported a range from 4.1 to 10.3 Brix in pumpkin. However, the previous research on soluble solids content of different smoothie formulations or products available on the market indicated similar results in the range from 8.6 to 15.4 [19,29,33].

### 2.2. Total Phenolic Content (tpc) in the Smoothies

The result showed that the total phenolic content (TPC) in produced smoothies ranged from 39.2 to 55.8 mg/100 g. The BCL smoothie had the highest total phenolic content followed by the BCW, the BPCW, and the PCH, while the PCL had the lowest total phenolic (Table 2). It could be concluded that total phenolic content in smoothies composed of banana and carrot was higher than in those composed of pumpkin and carrot. All raw materials used for smoothie production were a source of phenolics but, according to the literature, they contained different levels. Thus, the differences in total phenolic content could be attributed to the different ingredients used for smoothie formulation. Bashmil et al. [34] presented the total phenolic content from 38 to 128 mg of GAE/100 g for banana pulps depending on cultivars and maturity stage. Priori et al. [14] reported that the total phenolic content found in different evaluated pumpkins cultivars ranged from 26.31 to 79.89 mg/100 g. The richest source of phenolics could be the purple carrot. Leja et al. [35] found 311.5 mg/100 g of total phenolic content in its roots.

Smoothie products could be considered as a very rich source of phenolic compounds. For example, smoothies composed from orange juice, papaya juice, melon juice, carrot puree, and skimmed milk contained 45.6 mg GAE/100 mL [36], those composed from apple juice, orange juice, strawberry, whole apple (pulp+juice), and banana contained 44 mg GAE/100 mL [19], while 148.7 mg chlorogenic acid/100 g was found in the case of a smoothie consisting of orange carrot and water [37].

Phenolic compounds that came from smoothies could be easily available for biological action. As found by Ribeiro et al. [38] bioaccessibility of the smoothie phenolic compounds varied from 20 to 47% between gastric and intestinal digests. More than that, processing typically applied during smoothie preparation, such as heating and grinding, could increase the bioavailability of phenolic compounds. Unfortunately, up to now, there have been no recommendation for the amount of phenolic compounds consumptions. But some researchers suggest that a long–term intake of phenolics from diets could reduce the risk of certain diseases such as cancers, cardiovascular diseases, type 2 diabetes, osteoporosis, pancreatitis, etc. [39].

### 2.3. Anthocyanins Content

Anthocyanins belong to flavonoids. These compounds are present in many fruits and vegetables, and their products which give these fruits and vegetables their red, purple, and orange colors. In human nutrition, they play an important role as antioxidants, and they are used in the food industry as natural colorants. Anthocyanins are widely distributed in the human diet and, in the United States, 12.5 mg/day [40] are estimated to be consumed, while according to a European survey, anthocyanin consumption varies between 19.8 and 64.9 mg/day [41].

Total anthocyanin content ranged from 7.1 to 11.13 mg/100 g in all produced smoothies (Table 3). The BCL sample had the highest amount of total anthocyanin at 11.13 mg/100 g, followed by PCL and PCH (at about 10 mg/100 g), while the smoothies produced from banana and carrots without sugar had the lowest amount of total anthocyanins (BCW sample at 7.1 mg/100 g). González-Tejedor et al. [42] detected only 3.74 mg/100 g of anthocyanin in the purple smoothie that was composed of purple seedless grapes, cucumber, beet, and broccoli. The amount of anthocyanins in a smoothie produced by Keenan et al. [43] from strawberries, apples, apple juice, bananas, and oranges was 34.67 mg/100 g of dry weight.

Purple carrots have been reported as very good sources of anthocyanins, including acylated forms [10,11,44,45]. Anhocyanins were chromatogram recorded at 520 nm. Four different anthocyanins in were found in the examined smoothies. According to retention time, spectra characteristic, and data in the literature, four cyanidin derivatives are detected and two had acylated form with sinapic acid (compound 3) and ferulic acid (compound 4). The predominant compound 4 corresponds to cyanidin 3-*O*-feruloyl-(xylosyl-glucosyl-galactoside), which represents approximately 75–80% of all anthocyanin (Table 3).

In the present work, the only source of anthocyanins in smoothies was the purple carrot. Additionally, the amount of purple carrot used in the recipe was the same in all produced smoothies. Other factors, such as the presence of sugar or pH and heating could also affect anthocyanin content in the final product.

The most probable effect on anthocyanin content could be caused by the addition of sucrose. In the present study, the result showed that the smoothies produced with the addition of some sucrose (white sugar) had higher values of total anthocyanin content compared to those smoothies without sugar added. This is because the addition of sucrose could have had a significant protective effect on anthocyanins, as has been reported by Tsai et al. [46]. Additionally, Nikkhah et al. [47] reported a protective effect of sugar (sucrose 20%) on anthocyanin stability in berries, and they showed that the effect of added sugar on anthocyanin stability depends on its structure, concentration, and type of sugar.

Other studies suggest that acylated anthocyanins from purple carrot possess unusual stability when compared to unacylated compounds. The study of Sadilova et al. [48] observed that elderberry anthocyaninswere were sensitive to thermal treatment. After 3 h of heating, only 50% of elderberry pigments were retained at 95 °C. On the other hand, Kirca et al. [8] reported that half of initial content of anthocyanins from purple carrots juice degraded after 5 h of heating at 90 °C.

### 2.4. Carotenoids

The result of total carotenoids (Table 2) showed that BPCW smoothie produced from banana, pumpkin, and carrots had the highest amount of total carotenoids (108 µg/100 g), followed by PCL (72 µg/100 g) while BCL smoothie had the lowest amount of total carotenoids (11 µg/100 g).

According to chromatographic data, only α- and β-carotene were detected in produced smoothies despite other findings. In fresh banana, pumpkin, and purple carrot also lutein, lycopene, β-cryptoxanthin, violaxanthin, astaxanthin, antheraxanthin, and zeaxanthin were detected [49,50].

The smoothies which had pumpkins in their formulation showed at least two times higher content of total carotenoids than smoothies that had banana and carrots only. The value of total carotenoids obtained in this study was low compared to the result obtained by Nawirska-Olszanska et al. [51] who got the range from 4.9 to 7.4 mg/100 g fresh weight in pumpkin puree enriched with Japanese quince, cornelian cherry, strawberry, and apples. Balaswamy et al. [52] reported a range from 0.14 to 1.54 mg/100 g of total carotenoids in the smoothies made from mango, pineapple, sapota, pomegranate, and papaya.

### 2.5. Ascorbic Acid

Vitamin C (ascorbic acid) it is a water-soluble vitamin. Humans are not able to synthesize their own vitamin C, and it must be supplied from external sources, such as foods or supplements.

The result presented in Table 2 showed that the vitamin C content in the produced smoothies ranged from 0.25 to 0.41 mg/100 g. The PCL smoothie had the highest vitamin C content (0.41 mg/100 g), while the PBCW smoothie had the lowest vitamin C content (0.25 mg/100 g).

It should be admitted, however, that smoothies obtained in this study are not a significant source of vitamin C. A reason for this could be the low ascorbic acid content in the fruits and vegetables selected for smoothie formulation. Carrots have been reported to have only 4 to 29 mg of ascorbic acid per 100 g [53,54]. The ascorbic acid content of bananas varied from 2.5 to 17.5 mg/100 g [49]. Research on pumpkins revealed that the vitamin C content of this fruit ranged from 3 to 15 mg/100 g, depending on the variety and maturity stage [15].

Vitamin C is well known as the less stable nutrient during processing, and it is significantly affected by many factors, such as heat, oxygen, light, storage temperature, and storage time [55].Yadav [56] reported a significant reduction of ascorbic acid content in carrot and other fruit juice-blended nectar processed under a temperature of 80 °C for 5 min. Patras et al. [57] showed a significant reduction in ascorbic acid levels in the thermal processing of carrots purees that resulted in a 46% reduction, as compared to un-processed samples. During smoothie production, fruits were crushed (which could release endogenous enzymes) and/or heated, and these proccesses could strongly affect vitamin C content in the final product.

### 2.6. Antioxidant Activity

An antioxidant is any substance that delays or inhibits oxidative damage to a target molecule. The main property of an antioxidant is its ability to trap free radicals. Antioxidant activity, then, is the cumulative capacity of food components to scavenge free radicals [58]. In this study, free radical scavenging activity against ABTS^•+^ (2,2′-azinobis(3-ethylbenzthiazoline)-6-sulfonic acid was used.

The result showed that the antioxidant activity in the produced smoothies ranged from 4.3 to 6.2 µM TE/g (Table 2). The BCL smoothie had the highest antioxidant activity, followed by the BCW sample. The smoothies which contained pumpkin had the lowest antioxidant activity.

In this present study, the antioxidant activity values were quite high, and the use of different raw materials had an affect on antioxidant activity. In addition, the smoothies which had bananas in their formulation showed higher antioxidant activity than the pumpkin with carrots only. In our work, correlation between antioxidant activity and the total phenolic content (TPC) was calculated. The results obtained showed a high positive linear correlation between these parameters (Pearson correlation coefficient R = 0.937). Similar results have been well documented in other studies, and it has been reported that the antioxidant activity of many fruits was more closely correlated to total phenolic contents than to ascorbic acid and other compound content [59,60]. Jiratanan and Liu [61], in their research on antioxidant activity, concluded that depending on the particular product, processing parameters, and methods, thermal processing may enhance, reduce, or cause no change in total antioxidant activity.

It has been suggested that food processing such as cooking or grinding might improve the extractability of antioxidant compounds by breaking down the cell walls. Furthermore, the thermally processed smoothies had a high level of antioxidants after processing when compared to fresh or high hydrostatic pressure (HHP) processed smoothies. The antioxidant capacity of HHP processed smoothies decreased during storage, and the level of reduction was greater than in thermally treated samples. This could suggest that the enzymatic degradation systems play important role in antioxidant capacity deterioration. Thermal treatment could lead to the heat denaturation of enzymes, while HHP conditions could not inactivate them [62].

### 2.7. Color Attributes of Smoothies

Color is an important quality attribute of fresh and processed food products that determines the first impression and influences consumers’ choices, perceptions, and purchase behavior. The results of the instrumental color determination of the produced smoothies are presented in Table 4. The results showed that L* values of produced smoothies ranged from 12.9 to 26.5. The highest value of lightness (L*) was found in the BCW smoothie, and the darkest sample was the PCL smoothie. From the results obtained, the lightness of banana/carrot smoothies were about two times higher than the pumpkin/carrot smoothies. Positive a* values means that the hue of color is red, and these values ranged from 20.7 to 25.0. The red color of produced smoothies was attributed to the anthocyanin that comes from purple carrots. b* values of smoothies ranged from 2.9 to 11.7. The highest value was found in the PCH smoothie, and the lowest was found in the BCL smoothie. The presence of pumpkin in the smoothie formula increased the value of b* parameters in smoothies. Also, Buniowska et al. [63] observed a 16% higher value of the b* parameter in the smoothie which contained carrot juice, pumpkin, and mango, compared with a sample where papaya was used instead of pumpkin. Smoothies containing juçara, banana, and strawberry produced by Ribeiro et al. [23] were a little darker, but the intensity of red was at least two times lower compared to samples from our study.

### 2.8. Sensory Analysis

**Figure 1:**
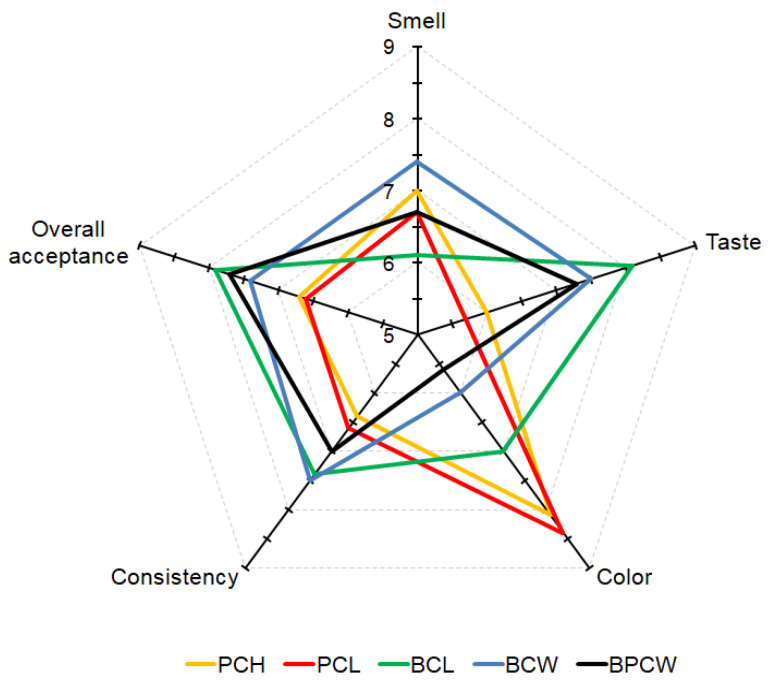
Sensory scores of different smoothies.

In Figure 1, sensory evaluation scores were presented. The studies showed that all products possessed good sensory attributes. The highest sensory score in terms of taste, consistency, and overall acceptance was found in smoothies composed of bananas and purple carrots (samples BCL and BCW). The smoothies produced from pumpkin and purple carrots (samples PCH and PCL) had higher scores in color than others, but their otherfeatures scored lower. Additionally, the BPCW sample obtained the lowest score in color, although this smoothie had good taste, consistency, and overall acceptance. The results also showed that in terms of overall acceptance, all produced smoothies were considered highly acceptable by the panelists. The BCL smoothie obtained the highest acceptance score (7.9), followed by the BPCW smoothie (7.7), while the PCL smoothie had the lowest score (6.6). These results showed that the materials incorporated into smoothie production have a great influence on consumer acceptability. Other research has suggested that soluble solids and the pH of smoothies had a strong effect on consumer acceptance. Smoothie products characterized by about 13 Brix degrees of soluble solids, and with pH values between 3.7 and 4.2, fit very well with consumers’ sensory preferences [33]. Sugars and acid ratio mainly influence the taste of the smoothie. Despite this, and the results of Uzodinma et al. [64], who had high scores in the sensory attributes for pineapple, watermelon, banana, and coconut smoothies without the addition of any external sweetener, low soluble solids smoothies (PCL and PCH) produced in our study obtained lower scores for taste.

## 3. Materials and Methods

### 3.1. Materials

Fresh pumpkin (*Cucurbita maxima* cv. Bambino) and purple carrots (*Daucus carota* ssp. sativus var. atrorubens Alef. cv. Purple Sun) were cultivated on a private farm located in the center of Poland (Sieradz district, Łódź province, Poland). Vegetables were harvested in October 2018. Unblemished and similar-sized pumpkins and carrots were selected and stored not longer than 4 weeks in a storeroom at about 4 °C until smoothie production. Ripe bananas, lemons, and white sugar were purchased from the local supermarket in Poznan, Poland.

All chemicals used were analytical or gradient grade (for HPLC) purity and provided by Poch (Polish Chemical Reagents, Gliwice, Poland) or Merck Kgaa (Darmstadt, Germany).

### 3.2. Smoothies Preparation

The smoothies’ production included two main steps, which were the pretreatment of raw materials, and blending. The steps for the pretreatment of raw materials were as follows: all ingredients were washed with tap water; purple carrots roots were peeled and chopped into about 2 cm pieces with a kitchen knife; pumpkin was peeled, and the seeds and the soft inner flesh parts were removed, before the pumpkin was chopped into about 2 cm cubic pieces; bananas were peeled by hand and chopped into about 2 cm pieces with a kitchen knife; the lemons were cut in half and the juice was squeezed using a portable citrus juicer. The second step was formulation and blending. The pumpkin, banana, purple carrot, lemon juice, water, and sugar were weighed according to the recipes presented in Table 5, before being placed in the Thermomix TM 3 device (Vorwerk SE & Co. KG, Wuppertal, Germany). The raw materials were ground and heated at 90 °C for 10 min to pasteurize the smoothie, and to produce a good, smooth texture. Five different products were obtained, as follows: pumpkin and purple carrot with high sugar (PCH); pumpkin and purple carrot with low sugar (PCL); banana and purple carrot with low sugar (BCL); banana and purple carrot without sugar (BCW); banana, pumpkin, and purple carrot without sugar (BPCW).

Smoothies were hot transferred to jars, closed with caps, and cooled in tap water. The sensory analysis and analysis of the color of the smoothies were performed a day after processing. For other measurements, smoothie samples were freeze-dried and ground into powder. Freeze drying was performed as follows: first of all, the samples were frozen in a low-temperature freezer (Arktico A/S, Esbjerg, Denmark) at −50 °C for 24 h, and then were subjected to freeze-drying in a freeze-dryer system, the FreeZone 6 dryer (Labconco, Kansas City, MO, USA), for 48 h. For the first 24 h, the temperature of the bulk tray dryer shelves was set at 4 °C for primary drying and then, for the next 24 h, it was set to 30 °C for secondary drying.

### 3.3. Determination of pH and Soluble Solids

The pH determination was conducted using a pH-meter Orion model 710A (Thermo Fisher Scientific, Waltham, MA, USA). The total soluble solids were determined in an optical refractometer, model HI96801 (Hanna Instruments, Woonsocket, RI, USA). The mean value was calculated from three measurements of each sample.

### 3.4. Total Phenolic Compounds (TPC) Extraction and Analysis

Phenolic compounds were extracted from freeze-dried samples with a mixture of methanol/water/acetic acid (25/24/1; *v*/*v*/*v*). The powdered sample (1 g) was mixed with extraction solvent (22 mL), and shaken for 1 h in a laboratory shaker. Then, the liquid was transferred to a 25 mL volumetric flask and filled up with the extraction solvent, which was followed by centrifugation for 5 min at 5000× *g*. The supernatant was collected in the tube for further analysis.

Total phenolic content (TPC) was analyzed using the Folin–Ciocalteu method [65]. A test tube was obtained, and 0.2 mL of the extract, 0.8 mL of distilled water, and 5 mL of Folin–Ciocalteu reagent (0.1 M) were added. The solution was roughly mixed for 2 min, then 4 mL of Na_2_CO_3_ solution (75 g/L) was added, and the reaction was carried out for 1 h at room temperature in darkness. Absorbance was read at 765 nm using a spectrophotometer UV-VIS 830 plus Metertech (Metertech Inc., Taiwan). All measurements were performed in triplicate, and the results were expressed as mg gallic acid equivalent (GAE) per 100 g of fresh weight of the sample.

### 3.5. Anthocyanins Analysis

The anthocyanin content of produced smoothies was determined by using the HPLC method described by Oszmiański & Sapis [66]. Phenolic extract was used for analysis. The HPLC system Agilent 1260 Infinity (Agilent Technologies Inc., Santa Clara, CA, USA) was used, equipped with a degasser, binary pump, autosampler holder, column holder, and a diode array detector (DAD). For chromatographic resolution, a Zorbax SB C-18 column with a diameter of 4.6 × 150 nm and 5 µm granulation was used. The chromatographic conditions were as follows: injection volume 20 µL; flow rate 1.0 mL/min; solvent A formic acid/water (1/9; *v*/*v*); solvent B: formic acid/acetonitrile/water (1/3/6; *v*/*v*/*v*). The elution gradient was linear as follows: from 0 to 15 min solvent B increased from 20 to 50%, from 16 to 20 min solvent B increased from 50 to 100%, from 20 to 21 min solvent B remained 100% and from 21 to 23 min solvent B decreased to 20%. The detector was set for scanning in the range of 400 to 700 nm. Quantification was performed at 520 nm and calculated as mg cyanidin-3-glucoside per 100 g of fresh weight of the sample.

### 3.6. Extraction and Analysis of Carotenoids

The carotenoids from each freeze-dried smoothie sample were extracted by using the accelerated solvent extractor Dionex ASE 350 (Thermo Fisher Scientific, Waltham, MA, USA) with acetone as the solvent. The extraction process was as follows: 1 g of each sample was weighed into extraction cells (34 mL), about 4 g of sand was added and mixed roughly. Pure acetone was used as the extraction solvent, with 3 cycles of 10 min for each sample. The pressure was 1500 psi and, after each cycle, the cells were washed with acetone. The process occurred at room temperature (23 °C). The obtained extract was evaporated and filled with acetone in a 10 mL volumetric flask.

The same HPLC system as for anthocyanin was used. The chromatographic conditions were as follows: injection volume 10 µL; flow rate: 0.5 mL/min; solvent A acetonitrile/0.05% triethylamine (*v*/*v*); solvent B methanol/ethyl acetate (11/9; *v*/*v*). The elution gradient was linear as follows: from 0 to 50 min solvent B increased from 5 to 40%, from 50 to 60 min solvent B increased from 40 to 80%, and from 60 to 75 min solvent B decreased from 80 to 5%. The detector was set for scanning in the range of 400 to 700 nm, while carotenoid quantification was performed at 454 nm. All measurements were performed in duplicate, and the results were expressed as µg of β-carotene/100 g of fresh weight of the sample.

### 3.7. Ascorbic Acid Extraction and Analysis

Extraction of ascorbic acid was prepared by mixing 1 g of each freeze-dried smoothie sample with about 22 mL of 1% meta-phosphoric acid. The mixture was shaken for 15 min in a laboratory shaker at room temperature. The solution was centrifuged at 4000× *g* for 10 min, and the supernatant was collected in a 50 mL volumetric flask and filled up with 1% meta-phosphoric acid. The obtained supernatant of 2 mL was mixed with 1 mL of 5% dithiothreitol and filled up to 10 mL with 1% meta-phosphoric acid. The sample was filtered through a 0.45 µm PTFE filter into the amber vials and analyzed by HPLC [67].

The same HPLC system as for anthocyanin was used. The chromatographic conditions were as follows: injection volume 10 µL; flow rate, 0.7 mL/min; solvent A 1 mM potassium dihydrogen phosphate in water; solvent B methanol. The elution gradient was linear as follows: from 0 to 6 min, solvent B increased from 5% to 22%, and from 6 to 15 min solvent B decreased from 22% to 5%. The detector was set for scanning in the range of 200 to 380 nm. Quantification was performed at 245 nm [67].

### 3.8. Antioxidant Activity

The antioxidant activity was determined using the ABTS^•+^ method, as described by Re et al. [68]. Phenolic extract was used for analysis. Absorbance of ABTS^•+^ solution in PBS pH 7.4 buffer was read after 6 min of incubation with the added sample or PBS buffer (control) at 30 °C at 734 nm, using a spectrophotometer Heλios α (Thermo Fisher Scientific, Waltham, MA, USA). From the absorbance measurement, the percentage of radical reduction of the extract was calculated from the following formula:

(1) $$\%\;of\;radical\;reduction=\frac{A_{c}-A_{s}}{A_{c}}\cdot 100\%$$

where A_c_—absorbance ABTS^•+^ solution of the control, A_s_—absorbance of ABTS^•+^ incubated with the sample. The percentage of radical reduction was plotted as a function of sample concentration or Trolox as a standard. The results were expressed as µM Trolox equivalents/g of fresh weight sample.

### 3.9. Instrumental Color Measurement

The color of the smoothie samples was measured using a spectrophotometer Konica Minolta 3600 d (Konica Minolta Co., Chiyoda, Japan). Color was expressed in CIE L*a*b* system coordinates. The smoothie samples were filled into a glass cuvette with an optical length of 10 mm, and this was placed over the aperture of the spectrophotometer. Then, reflectance color from the surface was measured using illuminant D65, at a 10° observer angle, with the specular component excluded. Three measurements for each sample were performed.

### 3.10. Sensory Evaluation

The sensory evaluation was conducted by a group of 7 panelists (4 women and 3 men, aged 20–45) with formal classroom training in sensory evaluation. To qualify, panelists had to be non-smokers, and had to have no allergy to any of the smoothie ingredients. All panelists work or study at Poznań University of Life Sciences and were previously involved in the sensory evaluation of fruits, vegetables, and their products. Samples were evaluated busing the 9-degree hedonic scale (9-extremely like; 1-extremely dislike). The assessment included the following quality attributes: smell, taste, color, consistency, and overall acceptance. All sensory tests were conducted in the room intended for sensory analysis, equipped with individual booths illuminated with normal lighting. All conditions in the testing area were controlled with minimum distractions to ensure panelists’ comfortable judgment.

### 3.11. Statistical Analysis

The analysis of variance (ANOVA) was used to determine the significance of the main effects. Tukey’s post-hoc test was used to determine differences between the mean values of multiple groups. Correlations were analyzed with Pearson’s test. Statistical significance was set at *p* < 0.05. The Statistica 13.1 software (TIBCO Software Inc., Palo Alto, CA, USA) and Excel 2010 (Microsoft Corporation, Redmond, WA, USA) were used for the calculations.

## 4. Conclusions

Smoothies produced from pumpkins, bananas, and purple carrots can be considered valuable products as sources of bioactive compounds and from sensory points of view. The findings revealed that the combination of these three ingredients contained a significant amount of total phenolic content (TPC), which ranged from 39.2 to 55.8 mg/100 g. The strong positive correlation between total phenolic content (TPC) and antioxidant activity was observed. The smoothies with pumpkin and purple carrots had higher total carotenoid content compared to those with bananas and purple carrots, whereas the smoothies that contained bananas in their formulation showed higher antioxidant activity compared to those with pumpkins and purple carrots only. The sensory panelists approved of all of the smoothies formulated. However, the smoothies produced from banana and purple carrots received excellent scores for smell, taste, consistency, and overall acceptance, while pumpkin and purple carrot smoothies received better scores for color. As a result, making smoothies from the selected ingredients could be convenient and an alternative to their commonly known products. However, more research is needed to check changes of bioactive compound content during the storage of smoothies. And to minimize the level of sugar added to the product, since excessive sugar consumption should be avoided from the nutritional point of view.

## Figures and Tables

**Table 1:** pH and total soluble solids of smoothies.

Sample	pH	Soluble Solids [%]
PCH	3.91 ± 0.01	10.5 ± 0.2
PCL	4.37 ± 0.01	7.9 ± 0.1
BCL	4.45 ± 0.01	16.7 ± 0.2
BCW	4.35 ± 0.01	12.7 ± 0.2
BPCW	4.45 ± 0.01	12.2 ± 0.1

**Table 2:** Total phenolic content, carotenoids, vitamin C content, and antioxidant activity of different smoothies.

Sample	Total Phenolic Content (TPC) (mg/100 g)	Sum of Carotenoids (µg/100 g)	Vitamin C (mg/100 g)	Antioxidant Activity (µM TE/g)
PCH	44.9 ± 2.9 ^ab^	62 ± 8 ^b^	0.34 ± 0.02 ^b^	4.3 ± 0.2 ^a^
PCL	39.2 ± 0.6 ^a^	72 ± 16 ^b^	0.41 ± 0.01 ^c^	4.3 ± 0.1 ^a^
BCL	55.8 ± 1.1 ^c^	11 ± 1 ^a^	0.27 ± 0.01 ^a^	6.2 ± 0.2 ^c^
BCW	47.4 ± 0.6 ^b^	45 ± 12 ^b^	0.31 ± 0.01 ^b^	5.2 ± 0.1 ^b^
BPCW	46.6 ± 2.1 ^b^	108 ± 10 ^c^	0.25 ± 0.01 ^a^	4.9 ± 0.1 ^b^

^a–c^—different letters in the same column indicate significant differences between data *p* < 0.05.

**Table 3:** Individual and the sum of anthocyanin content of different smoothies (mg/100 g; compound 1-cyanidin 3-xylosylglucosylgalactoside, compound 2-cyanidin 3-xylosylgalactoside, compound 3-sinapic acid derivative of cyanidin 3-xylosylglucosylgalactoside, compound 4-ferulic acid derivative of cyanidin 3-xylosylglucosylgalactoside).

Sample	Compound 1	Compound 2	Compound 3	Compound 4	Sum of Anthocyanins
PCH	0.63 ± 0.01	0.87 ± 0.02	1.01 ± 0.02	7.69 ± 0.17	10.2 ± 0.5 ^bc^
PCL	0.69 ± 0.01	0.93 ± 0.01	1.02 ± 0.01	7.75 ± 0.01	10.4 ± 0.3 ^cd^
BCL	0.64 ± 0.01	0.74 ± 0.01	0.70 ± 0.01	9.02 ± 0.02	11.1 ± 0.3 ^d^
BCW	0.47 ± 0.01	0.61 ± 0.01	0.45 ± 0.01	5.57 ± 0.08	7.1 ± 0.3 ^a^
BPCW	0.44 ± 0.01	0.54 ± 0.02	0.71 ± 0.01	6.31 ± 0.17	8.0 ± 0.5 ^ab^

^a–d^—different letters in the same column indicate significant differences between data *p* < 0.05.

**Table 4:** Color parameters of smoothies.

Sample	L*	a*	b*
PCH	14.7 ± 0.1 ^b^	25.0 ± 0.2 ^c^	11.7 ± 0.2 ^c^
PCL	12.9 ± 0.1 ^a^	20.7 ± 0.2 ^a^	7.7 ± 0.2 ^b^
BCL	22.9 ± 0.2 ^c^	24.5 ± 0.1 ^c^	2.9 ± 0.3 ^a^
BCW	26.5 ± 0.1 ^e^	22.3 ± 0.1 ^b^	3.4 ± 0.2 ^a^
BPCW	24.5 ± 0.1 ^d^	22.4 ± 0.1 ^b^	7.3 ± 0.2 ^b^

^a–e^—different letters in the same column indicate significant differences between data *p* < 0.05.

**Table 5:** Smoothie compositions (g/100 g).

Ingredient	PCH	PCL	BCL	BCW	BPCW
pumpkin	50	50	0	0	30
banana	0	0	40	44	40
purple carrot	10	10	10	10	10
lemon juice	4	2	2	3	3
sugar	6	4	4	0	0
water	30	34	44	43	17

## Data Availability

Data are available from the authors on request.

## Sample Availability

Samples of the compounds are not available from the authors.
